# Ligamentum Flavum Hematoma Accompanied by Intraspinal and Extraspinal Hematomas: A Case Report

**DOI:** 10.7759/cureus.38250

**Published:** 2023-04-28

**Authors:** Toshiaki Sugita, Shinji Tomari, Daichi Kitahara, Yasumasa Ito, Go Kato

**Affiliations:** 1 Orthopedic Surgery, Japanese Red Cross Fukuoka Hospital, Fukuoka, JPN; 2 Pathology, Japanese Red Cross Fukuoka Hospital, Fukuoka, JPN

**Keywords:** ligamentum flavum, extraspinal, intraspinal, hematoma, lumbar spine

## Abstract

Studies on ligamentum flavum hematoma (LFH) have been gradually increasing; however, no study has reported an LFH spreading to the intraspinal and extraspinal spaces. The purpose of this report is to discuss this rare condition and report that extraspinal hematoma can be formed by LFH. The authors present the case of a 78-year-old man presented with right L5 radiculopathy caused by a space-occupying lesion with intraspinal and extraspinal expansions at the L4-L5 vertebral levels demonstrated on MRI. We tentatively diagnosed these lesions as intraspinal and extraspinal hematomas originating from the ligamentum flavum based on the chronological changes seen on MRI and computed tomography-based needle biopsy. After the extirpation of these lesions, the symptoms were relieved. Three months later, the patient could walk without a cane. From the intraoperative findings and pathological examination, we concluded that the extraspinal hematoma in paravertebral muscle was caused by an LFH of unknown etiology. This case report describes the difficulty in diagnosing LFH accompanied by an extraspinal hematoma with wide-spreading expansion and highlights the usefulness of repetitive MRI over time in capturing chronological changes of the hematoma. As far as we know, this is the first study on an LFH accompanied by an extraspinal hematoma in the multifidus.

## Introduction

Lumbar cystic lesions in the epidural space, such as synovial or ganglion cysts related to the facet joints, can cause radiculopathies [1]. A less common type of cystic lesion is ligamentum flavum hematoma (LFH). Most LFH cases reported in the literature have affected elderly Asian males [2]; however, the detailed mechanism of the pathophysiology of LFH is still unknown. Studies have suggested that LFH could occur due to rupture of the proliferated capillary in the degenerative hypertrophied ligamentum flavum (LF) [3-5] or hemorrhage in the facet joint [6].

Magnetic resonance imaging (MRI) is the most useful imaging modality in diagnosing LFH. However, making an accurate preoperative diagnosis is difficult using single-time MRI because hemoglobin molecules in the hematoma chemically change over time, resulting in nonspecific MRI characteristics of LFH [4,7,8].

Here, we describe the case of a 78-year-old man with intraspinal and extraspinal space-occupying lesions (SOLs) at the lower lumbar spine revealed by MRI. The patient complained of lower leg pain and motor weakness. Initially, diagnosing these lesions preoperatively was difficult because of the wide-spreading expansion of the SOLs. A tentative diagnosis was made after several examinations including multiple MRIs. The final definitive diagnosis was made as hematomas originating from the degenerative LF according to the intraoperative findings and postoperative histopathological examination.

## Case presentation

History and presentation

**Figure 1:**
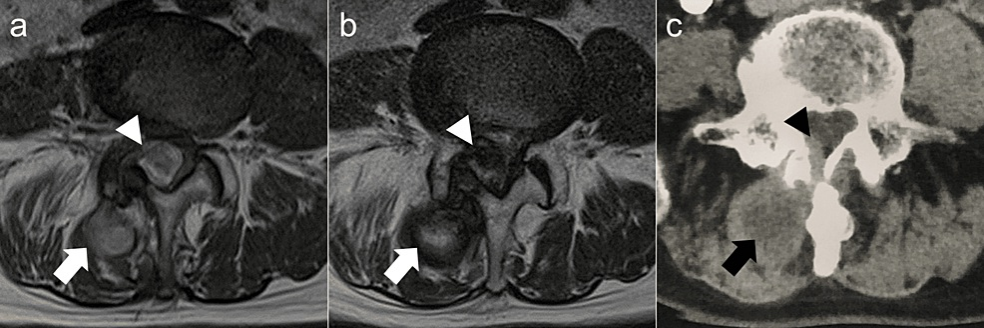
MRI and contrast-enhanced CT of intraspinal (arrowheads) and extraspinal space-occupying lesions (arrows) at the L4–L5 facet level on initial presentation. (a) Axial T1-weighted MRI shows an intraspinal, extradural SOL in the right dorsal aspect of the spinal canal and an extraspinal SOL in the multifidus. Both SOLs demonstrate isointensity to hyperintensity; (b) Axial T2-weighted MRI of these SOLs shows a partially hyperintense area surrounding a low-intensity area; (c) Contrast-enhanced CT of the intraspinal and extraspinal lesions at the L4–L5 facet level shows almost no enhancement. The sizes of the intraspinal and extraspinal SOLs are rostrocaudal 18 mm × dorsoventral 16 mm × mediolateral 30 mm and rostrocaudal 38 mm × dorsoventral 30 × mediolateral 30 mm, respectively. SOL: space-occupying lesion

A 78-year-old man presented with acute lower back pain and radicular pain in the right foot and was taken to our hospital in an ambulance. The patient reported that he had low back pain one month prior. The patient was known to have hypertension, paroxysmal atrial fibrillation, and Alzheimer’s disease and was on treatment with antihypertensive agents (angiotensin-converting-enzyme inhibitor and calcium-channel blocker), an anticoagulant agent (edoxaban tosilate hydrate), and an acetylcholinesterase inhibitor. While the activated partial thromboplastin time (PTT) and prothrombin time(PT)-international normalized ratio (INR) was within the normal range, the averaged PT-INR was slightly elevated at 1.3. The patient had no history of lumbar puncture, trauma, or surgery. On physical examination, he had a slight weakness of the right tibialis anterior muscle and pain and numbness in the buttocks and lateral lower leg. The straight leg raising test on the right side was positive at 30°. These findings indicated L5 radiculopathy. Plain radiography of the lumbar spine showed degenerative spondylosis and narrowing of the right L4-L5 disc height. Plain computed tomography (CT) demonstrated no calcification or ossification of the lumbar LF, except for a remarkable degenerative change of the right L4-L5 facet joint. MRI demonstrated an intraspinal and extradural SOL in the right dorsal aspect of the spinal canal and an extraspinal SOL in the right multifidus at the L4-L5 level. Furthermore, both SOLs demonstrated isointensity to hyperintensity on a T1-weighted image (Figure 1a) and partial hyperintensity area in the surrounding low-intensity area on a T2-weighted image (Figure 1b) and seemed to connect through L4-L5 LF or right facet joint. Because MRI demonstrated intraspinal and extraspinal wide-spreading SOLs, malignancy including metastatic spine tumor or extradural abscess was cited as a differential diagnosis besides hematomas. Neither tumor marker elevation in a blood test nor a contrast-enhanced lesion on whole-body CT was observed. In addition, almost no contrast enhancement was observed in both the intraspinal and extraspinal SOLs (Figure 1c) on CT.

**Figure 2:**
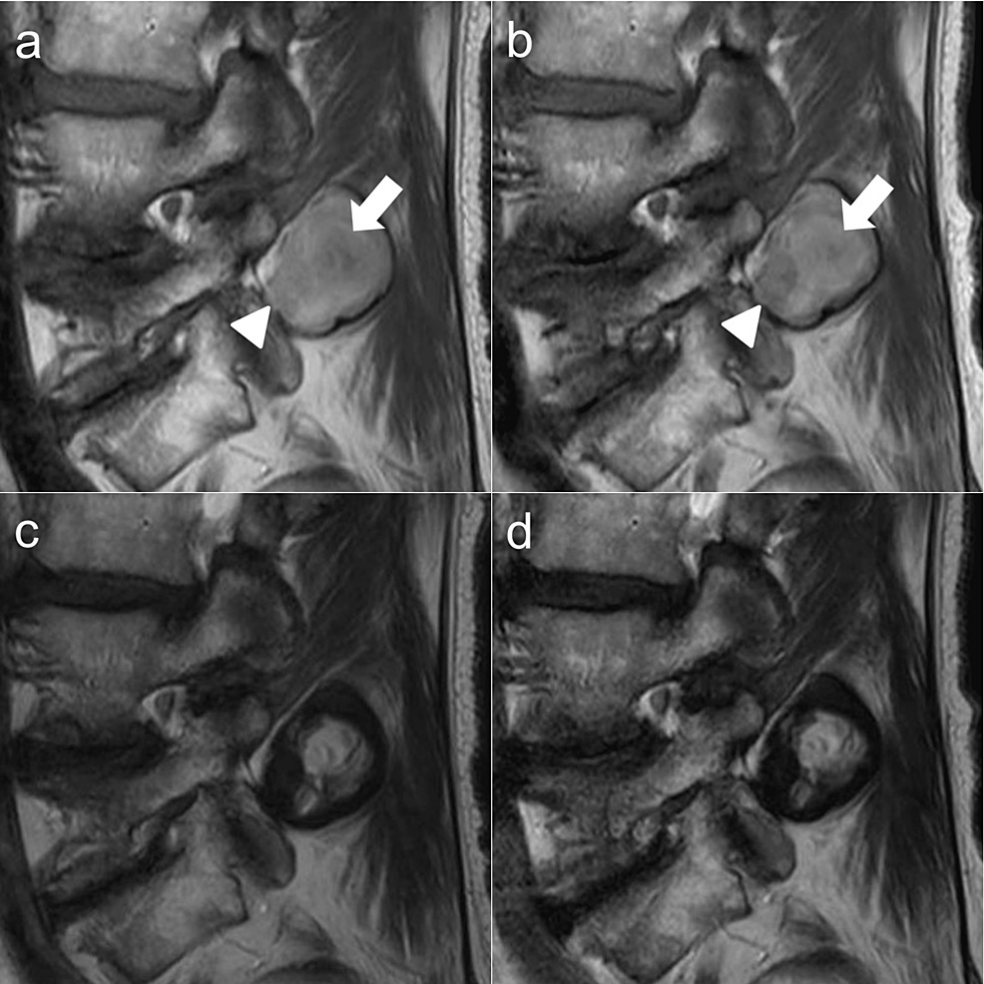
(a, b) Parasagittal MRI of extraspinal space-occupying lesions in the multifidus at the L4–L5 facet level on the 13th (left column) and 21st days of hospitalization (right column); (c, d) Sagittal T2-weighted MRI shows almost no signal changes. Signal changes of the dorsorostral region are indicated by arrows and those of the ventrocaudal area are indicated by arrowheads

Furthermore, CT-guided biopsy of a tissue sample from the extraspinal SOL showed no evidence of malignancy or infection. The possibility of LFH accompanied by an extraspinal hematoma was the final diagnosis; however, to our knowledge, no study has reported an LF hematoma spreading so widely as in this patient. We performed MRI multiple times to confirm the chronological changes in the images. Although no signal changes were observed on T2-weighted images, T1-weighted images showed evident signal changes in a part of the extraspinal SOL (Figure 2a-2d).

Therefore, we diagnosed these intraspinal and extraspinal SOLs as hematomas and performed surgery for the extirpation of both SOLs and decompression of the compressed lumbar dural sac.

Operation

**Figure 3:**
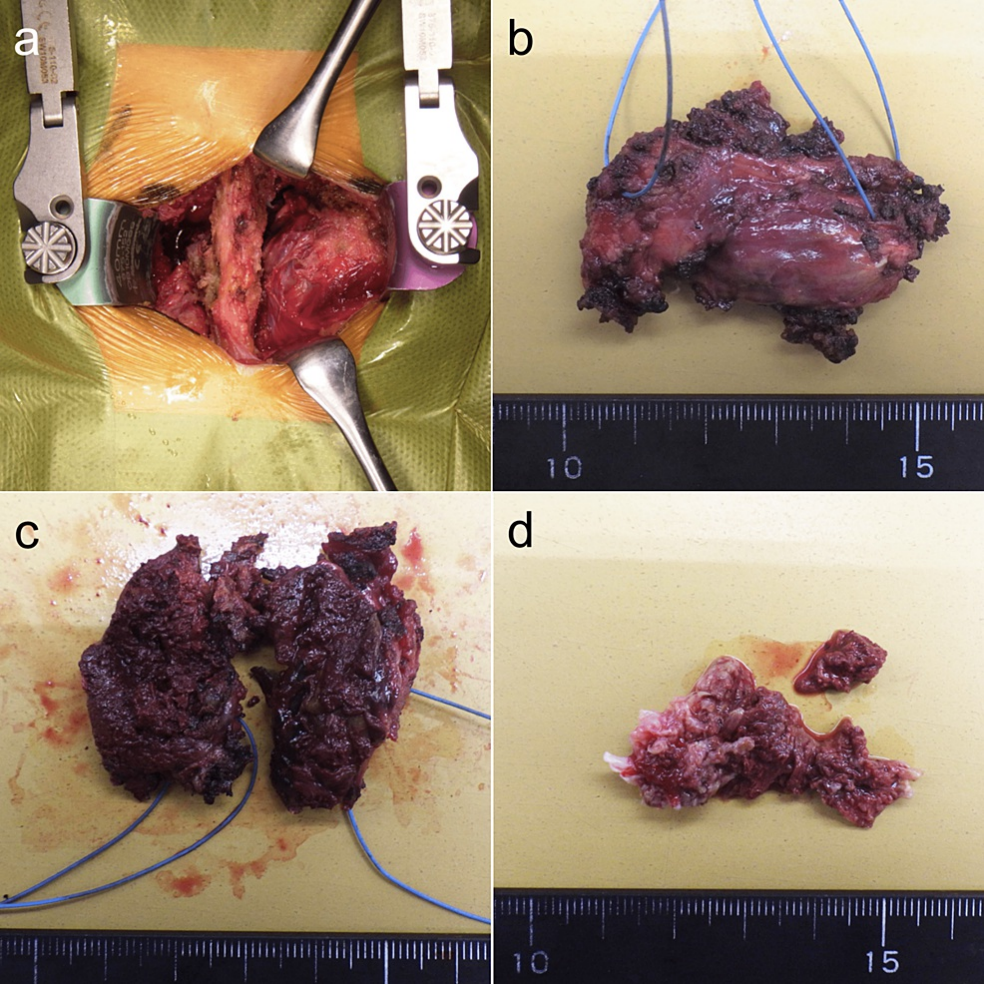
(a) An extraspinal space-occupying lesion (SOL) in the multifidus; (b) A resected extraspinal SOL; (c) Dark-red chronic hematoma was covered by a thin wall; (d) Brownish hematoma was attached to the hypertrophic ligamentum flavum.

We performed diagnostic and therapeutic surgery on the 23rd day of hospitalization. A midline incision was made from the L4 to L5 spinous processes. An elastic, hard, golf-ball-size SOL was exposed after the right multifidus was split bluntly (Figure 3a). The SOL was extirpated without rupture of the thin wall (Figure 3b). The ventral aspect of the SOL was attached to the LF at the L4-L5 level. Macroscopically, the cut surface of the SOL showed that it was filled with solid dark-red content (Figure 3c), suggesting that the SOL was a subacute or chronic hematoma. Then, flavectomy was performed, and the left-side dural sac was exposed after partial L4 laminectomy and bilateral L4-L5 medial facetectomy. The dural sac was compressed to the left side by the extradural and intraspinal SOL localized in the right aspect of the LF. A dark-red thick hematoma spouted out while breaking up the adhesions between the dura and extradural SOL. The L4-L5 facet joint capsule did not rupture. The LF exhibited hypertrophic changes, the hematoma included in the LF was extirpated en bloc (Figure 3d), and the right L5 nerve root and dural sac were decompressed. Although edoxaban tosylate hydrate was stopped a day before surgery, intraoperative oozing hemorrhage from the right aspect of the LF was relatively extensive (115 ml). A drain was placed and removed four days later.

Histopathological findings

**Figure 4:**
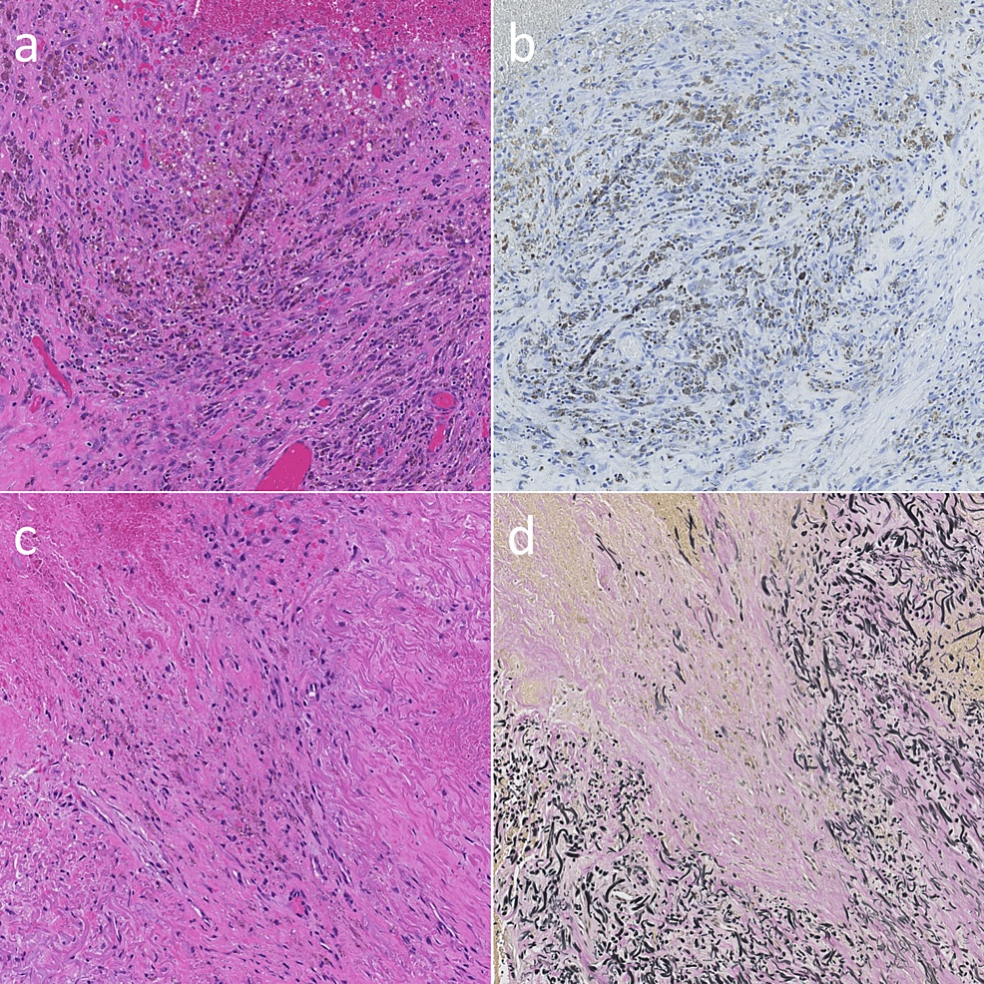
Histopathological findings of extraspinal SOL (a, b) and intraspinal and extradural SOL (c, d). (a) In the external wall of the extraspinal SOL, proliferation of granulation tissue and hemosiderin-laden macrophage deposits were observed; (b) Epithelial membrane antigen was negative, suggesting that the wall did not consist of synovial cells; (c) Besides hemosiderin-laden macrophage deposits, the proliferation of capillaries and erythrocytes outside these capillaries were observed; (d) In addition, elastica van Gieson staining of the extirpated LF revealed that elastic fiber bundles were irregularly arranged and fragmented with the expansion of the collagenous area. SOL: space-occupying lesion

In the external membrane of the extraspinal SOL, a proliferation of granulation tissue and hemosiderin-laden macrophage deposits were observed (Figure 4a). Epithelial membrane antigen was negative (Figure 4b), suggesting that the membrane did not consist of synovial cells. Besides hemosiderin-laden macrophage deposits, the proliferation of capillaries and erythrocytes outside these capillaries was observed (Figure 4c). In addition, elastica van Gieson staining of the extirpated LF revealed that elastic fiber bundles were irregularly arranged and fragmented with the expansion of the collagenous area (Figure 4d).

Postoperative course

**Figure 5:**
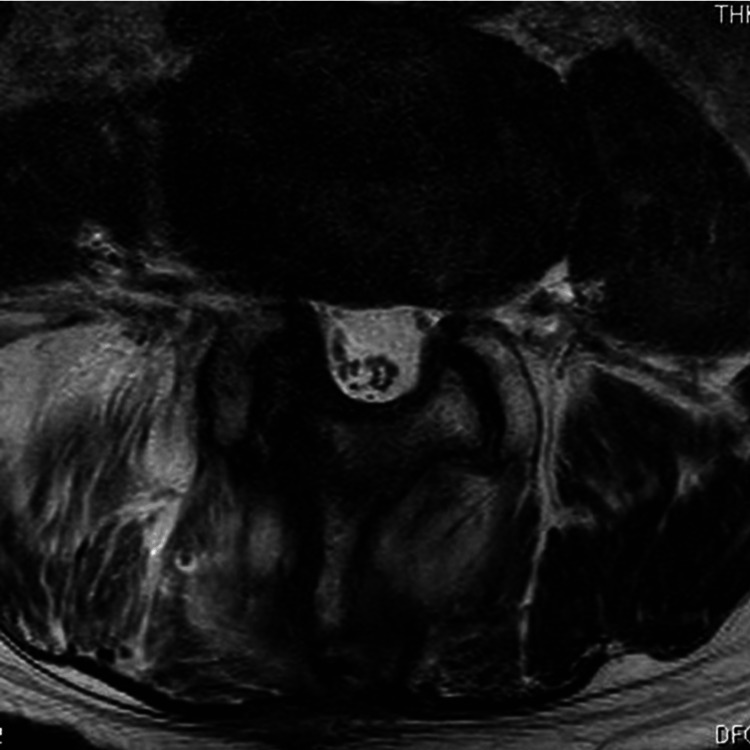
MRI at the L4–L5 facet level at three months after surgery. Axial T2-weighted MRI shows no hematoma recurrence

Postoperatively, right leg pain and numbness were relieved, and motor weakness gradually decreased. The anticoagulant agent was resumed a week after the operation. Three months later, the patient could walk without a cane. No hematoma recurrence was observed on MRI, and the dural sac at the L4-L5 level was well decompressed (Figure 5).

## Discussion

The number of case reports for LFH has increased since Sweasey and colleagues reported two lumbar LFH cases in 1992 [9]. As a whole, LFH mostly occurs in the lower lumbar spine of elderly Asian males as in the present case [2]. As far as we know, no studies in the literature have assessed the genetic factors for LFH in racial and ethnic differences, except for those describing LF ossification [10,11]. Thus, the genetic background for the high incidence rate of LFH in elderly Asian males is still unknown.

Regarding the inducement (external factor) of LFH in the lower thoracic spine of the mobile segment and lumbar spine, studies have speculated that the rupture of proliferated capillaries in the degenerated LF is induced by minor back trauma [12], exercise [13-16], physical exertion [3,5,9,13,15,16], or other unknown reasons [2,4,7,17-19] as also shown in this study. Furthermore, many studies have reported that patients with LFH had a medical history of hypertension (50% of patients with LFH whose past medical histories were described in the literature) [4,9,12,14,16,18,19] as our case did, and the elevation of intra-capillary pressure may induce the spontaneous rupture of the capillaries in the LF resulting in LFH. Our patient was prescribed an anticoagulant agent; however, only 3.4% of patients with LFH take anticoagulant agents and have coagulant factor abnormalities [17], suggesting that weakening of blood coagulability is not a strong risk factor for the occurrence of LFH. Although Nishida and colleagues suggested that LFH results from the expansion of hemorrhage in the facet joint, no sign of hemorrhage in the facet joint was observed in this study.

Regarding preoperative diagnosis, lumbar MRI showed that the LFH expanded to the intraspinal and extraspinal spaces in our patient. Since such an expansive hematoma originating from LFH has not been reported, we could not strongly suspect that both SOLs originated from the LFH; we inevitably cited malignancy and infection as differential diagnoses on the first visit.

MRI is the most useful imaging modality in diagnosing LFH. However, both T1- and T2-weighted MR images of hematomas change as hemoglobin molecules chemically change chronologically [7]. Furthermore, the LFH could be caused by repetitive bleeding and have a multistaged hematoma [13]; however, the onset of bleeding from LF is usually unknown and how many times bleeding in LF occurs cannot be determined. Moreover, these uncertainties can be involved in the complexity and variability of signal intensity of the lesion on MRI, and no specific pattern on contrast-enhanced MRI has been reported for this type of lesion: no enhancement, peripheral enhancement, and enhancement in a relatively large area in LFH [8]. Thus, the diagnosis of LFH is difficult using spinal MRI at the target level only once even if both plain and contrast-enhanced MRI are performed. Therefore, to diagnose LFH during the acute or subacute phase when hemoglobin molecules are still changing chemically, the usefulness of performing MRI several times as mentioned in the literature [8,18] should be emphasized, except for situations where repetitive MRI is contraindicated for rapid symptom progression. Moreover, LFH should be cited as the most likely diagnosis before malignancy, infection, or other cystic lesions if any patterns of chronological signal change on T1- or T2-weighted images are found.

In this study, several histopathological findings for the resected LF are commonly demonstrated in studies on LFH in the lower thoracic spine of the mobile segment and lumbar spine, such as elastic fiber degeneration [9,17], increase in collagen fiber [13,15], proliferation of capillaries [4,13,15], bleeding into the degenerated LF [4,5,9,13,17], and hemosiderin-laden macrophages [2,4,7,13], and these findings and intraoperative findings were solid evidence of LFH. However, it is particularly worth noting that in our case, the extraspinal hematoma was in contact with the LF and encapsulated by the granulation tissue formed by the hematoma with a high probability. This finding suggested that the extraspinal hematoma originated from the LFH.

In addition, the aforementioned histopathological findings of LFH resemble those reported in a study describing the histopathological findings of LF with calcium pyrophosphate crystal deposition [20]. In this study, microangiogenesis was observed around the area of the ruptured elastic fibers and collagen fibrils, and these changes were significant on the dorsal side (superficial layer) rather than the dural side (deep layer). According to this observation, it may be reasonable to think that bleeding and hematoma formation occur more frequently in the dorsal aspect of the LF (multifidus) rather than the ventral aspect when proliferated capillaries in the LF rupture. Moreover, if the extraspinal hematoma dorsal to LF occurs without an intraspinal hematoma following a neurological deficit, a spinal MRI will not be performed in clinical practice, resulting in the neglect of such an occult hematoma originating from the LF. Thus, it is possible that the incidence of extraspinal hematoma originating from the LF is much higher than that of clinically problematic intraspinal LFH.

 In our case, the neurological deficit was relatively mild, which allowed us to conduct non-invasive investigations, including obtaining repetitive MRI images at some intervals, and speculate that the SOLs were hematomas. However, when the neurological deficit is severe and/or progressive over time, it is better and more reasonable to opt for diagnostic surgery rather than non-invasive investigations. Therefore, our diagnostic approach may not be applicable in all the cases suspected of LFH. 

## Conclusions

This case report describes the difficulty in diagnosing LFH accompanied by an extraspinal hematoma with wide-spreading expansion and highlights the usefulness of repetitive MRI over time in capturing chronological changes of the hematoma. As far as we know, this is the first study on an LFH accompanied by an extraspinal hematoma in the multifidus. However, when considering the pathophysiological mechanism of LFH occurrence, it is possible that the occurrence rate of asymptomatic occult extraspinal hematomas originating from LFH is higher than we believe. Thus, this possibility would be noteworthy when diagnosing SOLs near the LF expanding into the intraspinal and extraspinal spaces.
